# Allogenic Transplantation of RPE Strips Lacking MHC Class II Can Avoid Rejection in Nonhuman Primate Eyes

**DOI:** 10.1167/iovs.66.6.53

**Published:** 2025-06-17

**Authors:** Atsuta Ozaki, Sunao Sugita, Masaaki Ishida, Mitsuhiro Nishida, Kyoko Iseki, Noriko Sakai, Naoko Hayashi, Takashi Shiina, Satoshi Yokota, Shin-ichiro Ito, Masashi Fujihara, Mineo Kondo, Masayo Takahashi, Yasuo Kurimoto, Michiko Mandai

**Affiliations:** 1 Kobe City Eye Hospital, Hyogo, Japan; 2 Cell and Gene Therapy in Ophthalmology Laboratory, BZP, RIKEN, Saitama, Japan; 3 Department of Ophthalmology, Mie University Graduate School of Medicine, Mie, Japan; 4 Vision Care, Hyogo, Japan; 5 Sugita Eye Clinic, Miyazaki, Japan; 6 Department of Ophthalmology, University of Toyama Graduate School of Medicine and Pharmaceutical Sciences, Toyama, Japan; 7 Tokai University School of Medicine, Kanagawa, Japan; 8 Laboratory for Animal Resources and Genetic Engineering, RIKEN Center for Biosystems Dynamics Research, Hyogo, Japan

**Keywords:** iPS cell, retinal pigment epithelium, transplantation, MHC class, rejection

## Abstract

**Purpose:**

We previously reported that suspensions of *CIITA*^−/−^ monkey induced pluripotent stem cell–derived retinal pigment epithelium (moiPSC-RPE) that lacks expression of major histocompatibility complex (MHC) class Ⅱ avoided rejection without systemic immunosuppression. RPE strips represent a novel graft formulation for RPE impairment diseases that may enable mass engraftment but potentially increase rejection risk. This study evaluated host immune responses to allogeneic *CIITA^+/+^* and *CIITA^−^^/^^−^* moiPSC-RPE strips in MHC-mismatched transplantation in monkey eyes with RPE damage, without systemic immunosuppression.

**Methods:**

RPE strips were generated from *CIITA^+/+^* and *CIITA^−^^/^^−^* moiPSC-RPE. Following RPE ablation using a microsecond pulse laser, two RPE strips were transplanted into each eye at 1- to 3-week intervals. One monkey received *CIITA^+/+^* moiPSC-RPE strips, while another received *CIITA^−^^/^^−^* moiPSC-RPE strips. Graft status was monitored through routine ophthalmic examinations, and immunohistologic evaluation was conducted after 5 months.

**Results:**

Fluorescein angiography revealed evident leakage in eyes with *CIITA^+/+^* RPE strips beginning 1 to 2 months posttransplantation, with optical coherence tomography showing bulging at the graft site, indicating immune cell accumulation. Immunostaining confirmed the presence of immune cells (CD4, CD8, Iba1, IFN-γ, MHC class II, and CD20) at *CIITA^+/+^* RPE strip grafts. In contrast, *CIITA^−^^/^^−^* RPE strip transplants showed no signs of rejection in either eye over 5 months and minimal immune cell presence at the graft site.

**Conclusions:**

Allogeneic transplantation of *CIITA^−^^/^^−^* RPE strips may reduce rejection risk in RPE-ablated disease model eyes, suggesting potential clinical benefits of MHC class II–deficient RPE grafts, particularly for diseases with inflammatory pathology.

Impairment of the retinal pigment epithelium (RPE), as seen in age-related macular degeneration (AMD) and inherited retinal diseases, leads to photoreceptor cell loss. Transplantation of healthy RPE cells represents a promising therapeutic strategy. Two major formulations exist for RPE transplants: cell suspensions and sheets. While cell suspensions are easily injectable, they often disperse in the vitreous. Conversely, RPE sheets ensure targeted delivery but require large, invasive incisions. To address these limitations, we recently developed pluripotent cell-derived RPE strips that are formed by quick self-aggregation of RPE cells without using any artificial scaffold by seeding cells in narrow grooves for 2-day incubation.^1^ Our transplantation of human induced pluripotent stem cell–derived RPE (hiPSC-RPE) strips into four monkeys demonstrated minimal backflow leakage and successful expansion with minimally invasive surgery.^2^ However, histologic analysis revealed immune cell accumulation at the graft site despite cyclosporine administration. Additionally, in our recently conducted clinical trial of RPE strip transplantation, we observed one case of mild rejection following discontinuation of oral cyclosporine, which was administered for the first 6 months in all patients (jRCTa050210178).^3^

While the subretinal space, the target site for RPE cell transplantation, is considered immune-privileged,^4^ hiPSC-derived RPE cells express major histocompatibility complex (MHC) class II under inflammatory conditions, potentially increasing transplant immunogenicity.^5^ We previously conducted clinical studies using autologous or human leukocyte antigen (HLA)–matched hiPSC-derived RPE cells in patients with neovascular AMD without systemic immunosuppression.^6^^–^^8^ However, such case-dependent cell production remains costly and time-consuming.

Recent advances in MHC-related gene editing of iPSCs offer promising options for generating low-immunogenicity grafts.^9^^–^^11^ Petrus-Reurer et al.^9^ achieved this by generating human embryonic stem cell lines lacking MHC class I or II through *B2M* or *CIITA* gene targeting. Ishida et al.^11^ developed monkey iPSC (moiPSC) lines lacking the *CIITA* gene, the transactivator of MHC class II, which differentiate into RPE cells that do not express MHC class II after interferon gamma (IFN-γ) stimulation. Notably, both *CIITA^−^^/^^−^* and *CIITA^+/+^* moiPSC-RPE cells demonstrated similar properties in phagocytosis function and expression of RPE-specific markers and proteins.

In our previous study, we found that the monkey Embryonic Stem Cell (moESC)-derived RPE sheet that happened to lack MHC class II did not evoke any immune reaction for 2 years.^11^ Then we transplanted *CIITA^+/+^* and *CIITA^−^^/^^−^* moiPSC-RPE suspensions into MHC-mismatched monkey eyes. While *CIITA^−^^/^^−^* moiPSC-RPE suspensions showed no rejection for 6 months without systemic immunosuppression, *CIITA^+/+^* moiPSC-RPE suspensions exhibited minimal rejection. However, the scattered engraftment pattern of RPE cells made the effectiveness of *CIITA^−^^/^^−^* moiPSC-RPE cells somewhat ambiguous.^11^

Although iPSC-RPE strips enable minimally invasive surgery and stable delivery of substantial RPE cells that can expand sheet-like over damaged areas,^2^ larger cell aggregates may trigger immune responses, particularly in inflammatory conditions such as neovascular AMD.^8^^,^^12^^,^^13^ In this study, we evaluated immune responses to allogenic moiPSC-RPE strips by transplanting two RPE strips per eye at 1- to 3-week intervals, following local RPE ablation using a microsecond pulse laser. One monkey received *CIITA^+/+^* moiPSC-RPE strips, while another received *CIITA^−^^/^^−^* moiPSC-RPE strips. We assessed immune responses through in vivo ophthalmic imaging and immunohistochemical analysis of postmortem eyes.

## Methods

All animal experiments were conducted in accordance with local guidelines and the ARVO Statement on the Use of Animals in Ophthalmic and Vision Research. All of the experimental protocols were approved by the Institutional Animal Care and Use Committee of RIKEN Kobe Branch.

### Animals

Two cynomolgus monkeys were used for transplantation (monkey 1 and monkey 2), and paraffin sections were used from the cynomolgus monkey that had received the same microsecond pulse laser treatment in our previous study.^2^ All monkeys were obtained from Eve Bioscience (Hashimoto, Wakayama, Japan). The ages and transplantation features are documented in Supplementary Table S1. Monkey 1 received *CIITA^+/^*^+^ moiPSC-derived RPE strips, while monkey 2 received *CIITA^−^^/^^−^* moiPSC-derived RPE strips.

### MHC Typing for Monkeys

Genotyping of MHC class Ⅰ and Ⅱ haplotypes in the cynomolgus monkeys was determined by the Sanger sequencing method and high-resolution pyrosequencing.^14^ The results are listed in Supplementary Table S2.

### Differentiation of Monkey iPSC-Derived RPE Cells

The moiPSC line 1121A1, derived from the HT-1 MHC homozygote cynomolgus monkey,^15^ was maintained on iMatrix511 (Nippi, Tokyo, Japan) with StemFit AK02N medium (AJINOMOTO, Tokyo, Japan), with the medium changed every 2 to 3 days. For differentiation, the moiPSCs were cultured on laminin-coated dishes in medium containing 20% KSR supplemented with CKI-7 (CKI) (3 µM; Sigma-Aldrich, St. Louis, MO, USA), SB431542 (SB) (5 µM; Sigma-Aldrich, St, Louis, MO, USA), and Y-27632 (Y) (10 µM; Wako).^11^ The differentiation medium was changed sequentially: 20% KSR with Y + SB + CKI for 3 days, 15% KSR with Y + SB + CKI for 4 days, 10% KSR with Y + SB + CKI for 5 days, and 10% KSR for 13 days. The cells were then maintained in RPE maintenance medium until RPE differentiation. Medium was changed every 2 to 3 days throughout the differentiation process.

### Preparation of MoiPSC-Derived RPE Strips

MoiPSC-RPE cells were cultured for 2 weeks after thawing. RPE strips were generated in 2-mm-wide grooves using a previously described culture device.^1^^,^^2^ A suspension of 2.0 × 10⁶ RPE cells was seeded in grooves containing RPE maintenance medium supplemented with 10 µM Y-27632. The cells were cultured for 2 days.

### Laser-Induced RPE Damage Model

RPE was selectively damaged using a Micropulse laser (IQ 577; IRIDEX Corporation, Mountain View, CA, USA) as previously described.^2^ Using a laser contact lens (macular lens 901; Haag-Streit, Köniz, Switzerland) that provides a magnification of 0.96×, the pattern scan consisted of nine spots arranged in a 3 × 3 grid. The laser parameters included a spot spacing of 0 µm, a spot size of 100 µm, and a duration of 15 ms. The micropulse power was set to 50% of the power at which white color appeared. The duty cycle was 1% (0.05-ms pulse on and 4.9-ms pulse off). The RPE damage was confirmed by fluorescein angiography (FA) and fundus autofluorescence (FAF).

### Transplantation of MoiPSC-RPE Strips Into Subretinal Space of Monkeys

The surgery was performed following the procedure described in our previous report.^2^ After routine vitrectomy with posterior vitreous detachment, retinal detachment was induced at the laser-treated area using a 38-gauge cannula (PolyTip Cannula 38G; MedOne Surgical, Sarasota, FL, USA) with Opti-MEM (Thermo Fisher Scientific, Waltham, MA, USA) with ROCK inhibitor Y-27632 (10 µM). A secondary retinal hole was created within the detached retina at the opposite side for pressure escape to prevent graft strip backflow. Two RPE strips were aspirated into a customized 31-gauge cannula (Extendable PolyTip Cannula 25/31 GLT; MedOne Surgical, Sarasota, FL, USA) with approximately 3 µL SHELLGAN 0.5 (Santen Pharmaceutical, Osaka, Japan)/Opti-MEM solution (1:6 ratio) and injected into the bleb. Perfluoro-n-octane (Perfluoron; Alcon, Fort Worth, TX, USA) was injected to flatten the retinal detachment by pressing out the subretinal fluid, followed by fluid-air exchange and silicone oil (SILIKON 1000; Alcon, Fort Worth, TX, USA) injection. Intravitreal triamcinolone acetonide (20 mg/0.05 mL; Maqaid, Wakamoto, Tokyo, Japan) was administered after oil replacement. Silicon oil was removed 1 month after transplantation.

### In Vivo Imaging After Transplantation

The RPE strips were monitored using fundus image, FAF, FA (CX-1, Canon, Tokyo, Japan), and optical coherence tomography (OCT) (RS-3000; NIDEK, Gamogori, Aichi, Japan) at 2 weeks, 1 month, 2 months, 3 months, 4 months, and 5 months after transplantation. The graft area was measured using ImageJ (ImageJ bundled with Java 8; National Institutes of Health, Bethesda, MD, USA) with reference to AF images.

### Immunohistochemistry

The monkey eyes were extracted 5 months after transplantation. The eyes were fixed with methanol and formaldehyde (SUPERFIX; Kurabo, Osaka, Japan) for 7 days after corneal limbus puncture. The fixed tissue was embedded in paraffin (Sigma-Aldrich) and sectioned into 10-µm-thick slices using an automated slide preparation system (AS-200; Kurabo, Osaka, Japan). The sections were deparaffinized, and antigen retrieval was performed using either of three methods: (1) microwave treatment in 10 mM citrate buffer, pH 6.0, 100°C for 20 minutes; (2) an autoclave treatment in 10 mM citrate buffer, pH 6.0, 120°C for 20 minutes; or (3) an enzyme treatment in 0.05% Trypsin, 37°C for 10 minutes. The slides were blocked with a blocking solution for 1 hour at room temperature, then incubated with primary antibodies (Supplementary Table S3) overnight at 4°C. This was followed by secondary antibody incubation for 1 hour at room temperature. The samples were imaged using LSM700 (Zeiss, Jena, Germany) and SP8 (Leica, Wetzlar, Germany).

## Results

### Transplantation of *CIITA*^+/+^ and *CIITA*^−/−^ MoiPSC-RPE Strips

**Figure 1.**
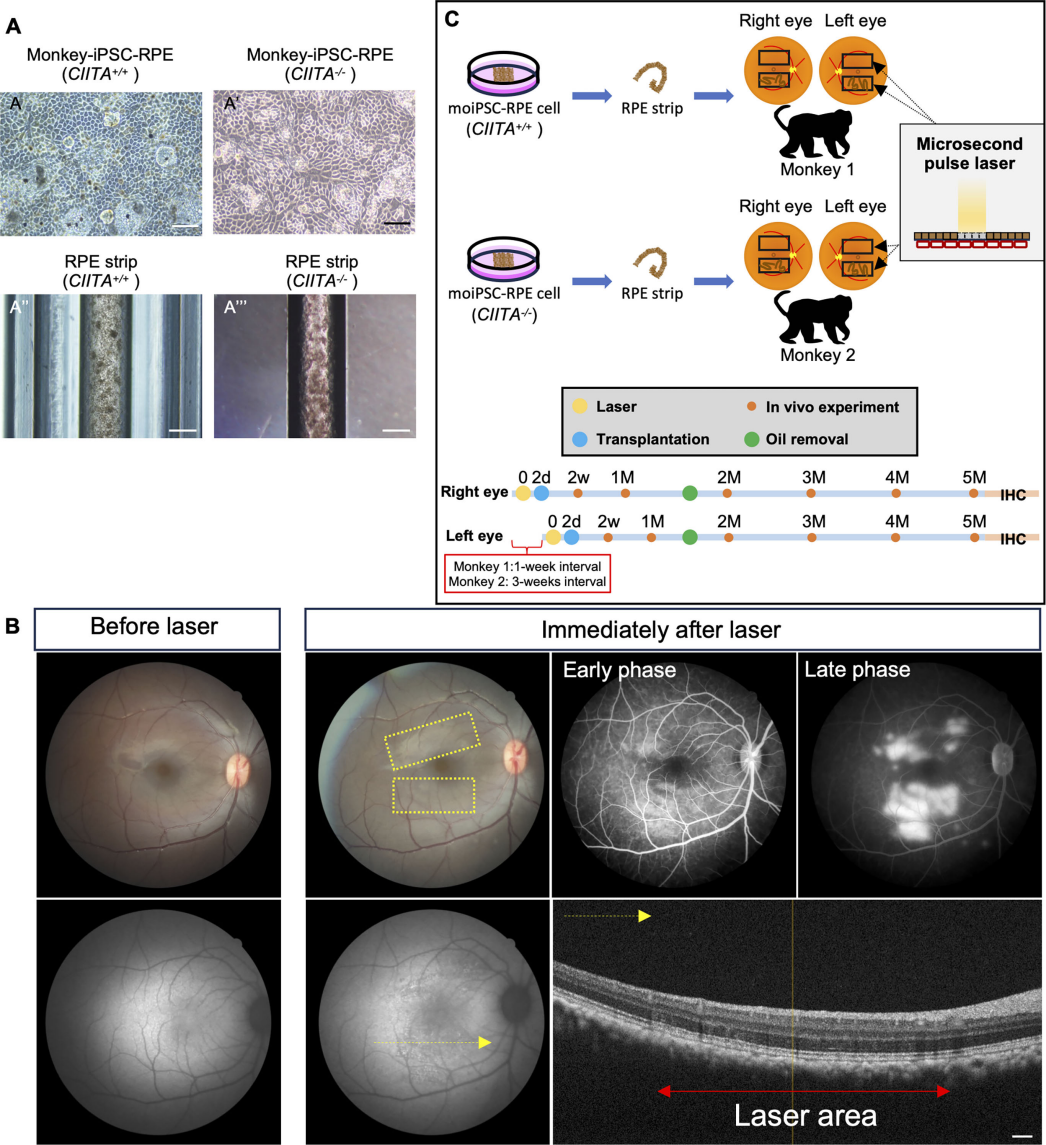
Preparation for RPE strips, micropulse laser for RPE damage, and experimental design. (**A–A′**) *CIITA^+/+^* (**A**) and *CIITA^−^^/^^−^* (**A′**) moiPSC-derived RPE cells showed polygonal morphology and melanin content. RPE strips were generated by seeding *CIITA*^+/+^ (**A′′**) and *CIITA*^−/−^ (**A′′′**) cells onto grooves and culturing for 2 days, forming strip-like structures. (**B**) Micropulse laser treatment was conducted 2 days before transplantation in each monkey eye. RPE damage was confirmed by pale white area in a fundus image (*upper left*, *yellow rectangle*), hypofluorescence in FAF (*lower left*), slight leakage in the late phase of FA (*upper right*), and a rough interdigitation zone and RPE–Bruch's membrane complex in OCT (*lower right*, *red arrow*). (**C**) Experimental design. Four eyes from two monkeys received RPE strip transplants. Monkey 1 received *CIITA^+/+^* RPE strips in both eyes, and monkey 2 received *CIITA^−^^/^^−^* RPE strips in both eyes. Two days before transplantation, regions above and below the fovea were laser-treated, followed by transplantation of two RPE strips below the fovea. Transplantation was first performed in the right eye and then in the left eye after 1 week in monkey 1 and after 3 weeks in monkey 2. The monkeys were monitored using fundus image, FAF, FA, and OCT at 2 weeks, 1 month, and monthly thereafter until euthanization at 5 months posttransplantation for immunohistochemical evaluation. Time points are indicated as d (day), w (week), and M (month). *Scale bar*: (**A**, **A**′) 10 µm, (**A′′**, **A′′′**) 50 µm, (**B**) 300 µm.

We used the RPE cells differentiated from the same *CIITA^+/+^* and *CIITA^−^^/^^−^* moiPSC (1121A1) stocks, which were characterized in our previous study (Figs. 1A–A′).^11^ Both *CIITA^+/+^* and *CIITA^−^^/^^−^* moiPSC-RPEs formed strip aggregates in 2 days (Figs. 1A′′–A′′′). We performed microsecond pulse laser treatment to the segments superior and inferior to the fovea 2 days before transplantation, as previously reported.^2^ Immediately following the laser treatment, we confirmed damage to the RPE by the presence of following features: (1) pale white areas in the fundus image, (2) hypofluorescence in FAF, (3) slight leakage in FA, and (4) a rough interdigitation zone and RPE–Bruch's membrane complex in OCT (Fig. 1B). We then successfully transplanted two moiPSC-RPE strips into the lower RPE damaged site in the right eye of two monkeys 2 days after laser treatment; one monkey received *CIITA*^+/+^ RPEs (monkey 1), and the other received *CIITA^−^^/^^−^* RPEs (monkey 2). For the left eyes, monkey 1 received *CIITA*^+/+^ RPE strips 1 week later, while monkey 2 received *CIITA^−^^/^^−^* RPE strips 3 weeks later (Fig. 1C).

### *CIITA*^−/−^ RPE Strips Showed Minimal Signs of Rejection in the In Vivo Ophthalmic Examination

**Figure 2.**
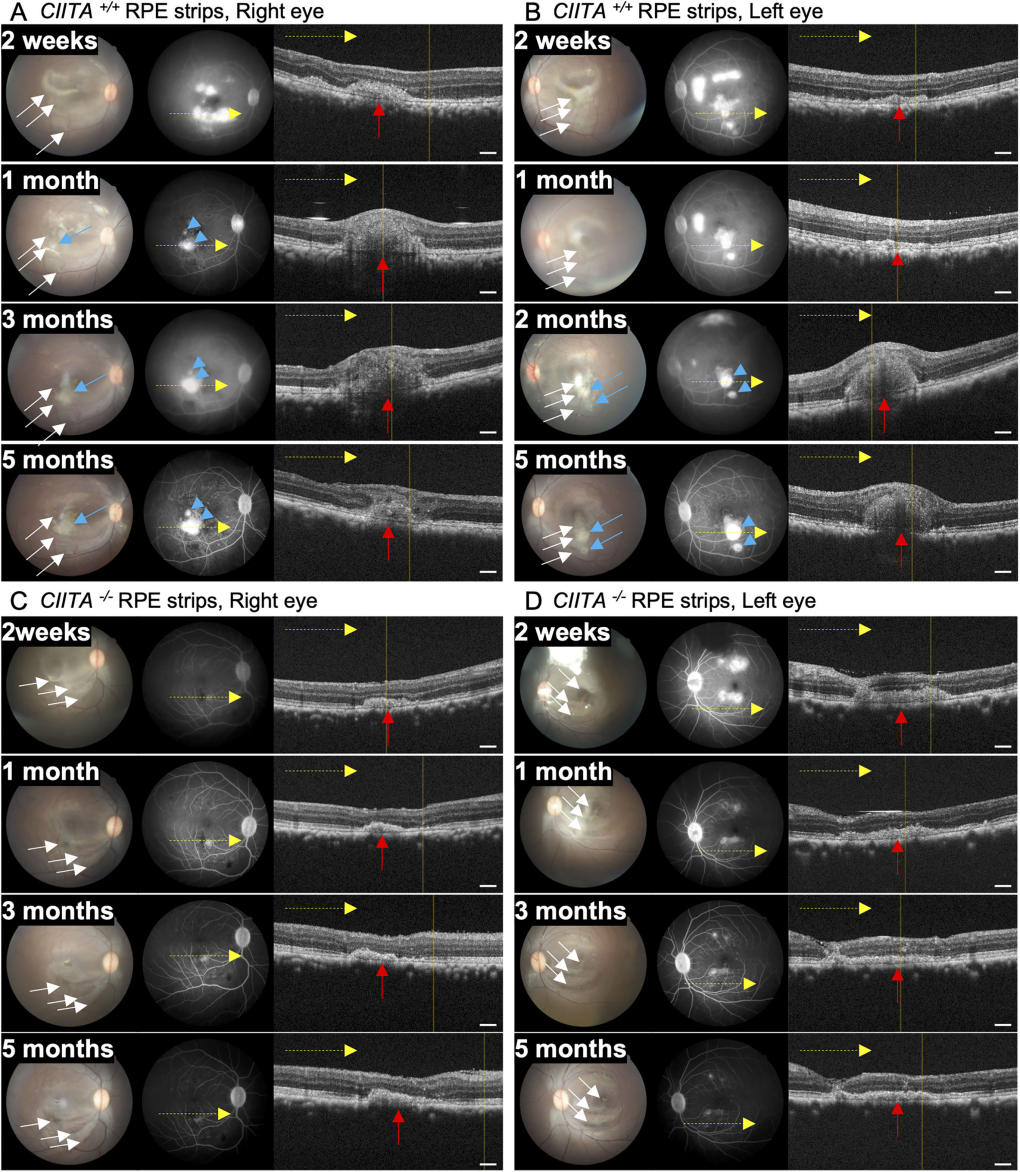
Distinct immune responses between *CIITA^+/+^* and *CIITA^−^^/^^−^* RPE strip transplants in nonhuman primates. (**A**, **B**) Representative images from both eyes of monkey 1 transplanted with *CIITA^+/+^* moiPSC-derived RPE strips. Transplanted RPE strips were initially slightly visible in fundus images (*white arrows*). White material appeared around the transplanted area at 1 to 2 months posttransplantation (*blue arrow*) and expanded through 5 months, likely indicating immune cell infiltration. This was accompanied by evident leakage in FA (*blue arrowhead*) and a bulge at the graft site in OCT (*red arrows*). (**C**, **D**) Representative images from eyes of monkey 2 transplanted with *CIITA^−^^/^^−^* moiPSC-derived RPE strips. The grafts were clearly visible and gradually became pigmented on the fundus image (*white arrows*). The graft RPE is indicated by *red arrows*. These images show no signs of immune rejection for 5 months. *Scale bar*: (**A–D**) 300 µm.

Fundus images revealed a white mass at the transplant site in the right eye of monkey 1 with *CIITA^+/+^* RPE strip transplantation 1 month after transplantation, which gradually expanded over time (Fig. 2A, Supplementary Fig. S1). FA images demonstrated a leakage from laser-treated area in the late phase 2 weeks after transplantation, which diminished 1 month after transplantation. However, evident leakage from the transplantation area was observed starting 1 month after transplantation (Fig. 2A, Supplementary Fig. S1). OCT revealed a bulge at the graft site, indicative of cellular infiltration. In the left eye with *CIITA^+/+^* RPE strips, similar rejection signs were evident 2 months after transplantation (Fig. 2B, Supplementary Fig. S1). In contrast, there were no rejection signs in either eye of monkey 2, which received *CIITA^−^^/^^−^* RPE strips. The grafts survived in the subretinal space through 5 months (Figs. 1C, 1D). During this time, *CIITA^−^^/^^−^* RPE strips gradually acquired dark pigmentation (Supplementary Figs. S2A, S2B). We then evaluated the expansion of the grafted RPEs, which was well identified in monkey 2 with *CIITA^−^^/^^−^* RPE strips, but not in monkey 1, in which cell infiltration disabled accurate measurement. *CIITA^−^^/^^−^* RPE strips gradually expanded from 2 weeks to 5 months in both eyes (Supplementary Figs. S2A–C). We quantitatively evaluated the change in maximal *CIITA^−^^/^^−^* RPE strip height in each eye (Supplementary Figs. S2A, S2B, S2D). Consistent with our previous report,^2^ the height of the RPE strips gradually decreased.

### *CIITA*^−/−^ RPE Strips Did not Evoke Immunologic Responses by Immunohistochemistry

**Figure 3.**
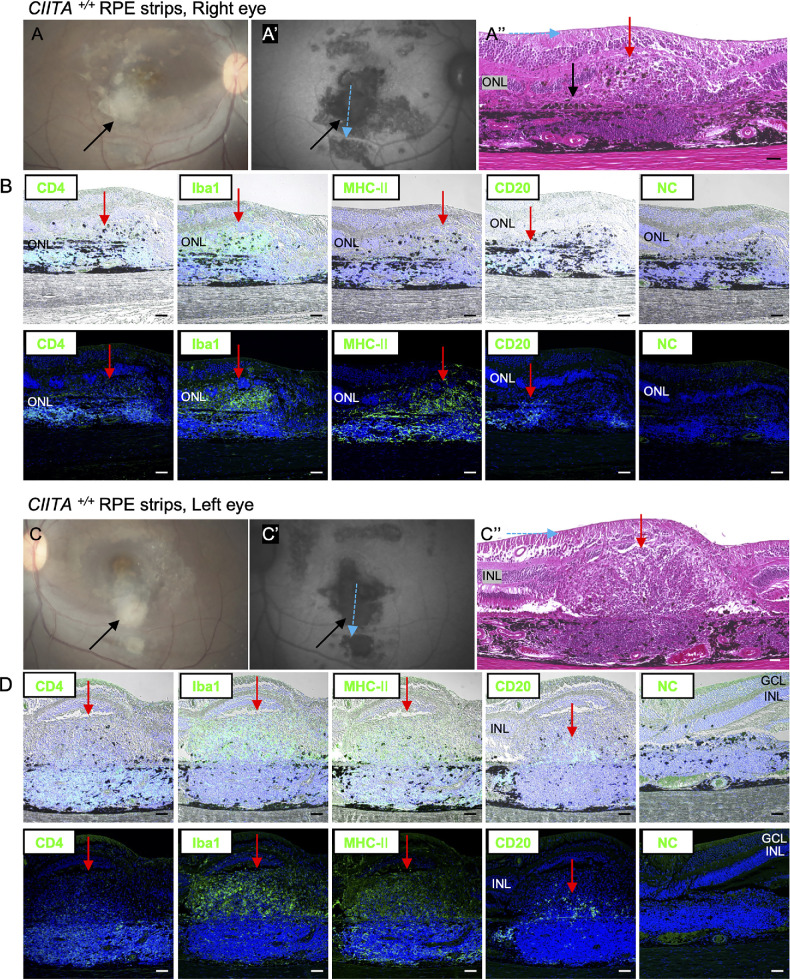
Histologic evidence of immune rejection in *CIITA^+/+^* RPE transplants (monkey 1, both eyes). (**A–A**′′, **C–C**′′) Fundus (**A**, **C**) and FAF images (**A**′, **C**′) of *CIITA^+/+^* RPE transplants in the right eye (**A**) and left eye (**C**). Transplanted RPE cells are indicated by *black arrows*. The *blue arrow* denotes the sectioning orientation. H&E staining of *CIITA^+/+^* RPE strips in the right eye reveals probable grafted RPE cells (*black arrow*) and cellular accumulation (*red arrow*), consistent with immune cell infiltration. The neural retina appears disorganized due to rejection. (**B**, **D**) IHC analysis demonstrates the presence of inflammatory cells (CD4^+^, Iba1^+^, MHC class II^+^, CD20^+^) in the right (**B**) and left (**D**) eyes. The *upper panels* show brightfield images overlaid with fluorescent images, and the *lower panels* show fluorescent images. The *red arrows* show accumulated immune cells. NC refers to the negative control with no primary antibody. *Scale bar*: (**A–D**) 50 µm. CD, cluster of differentiation; H&E, hematoxylin and eosin.

**Figure 4.**
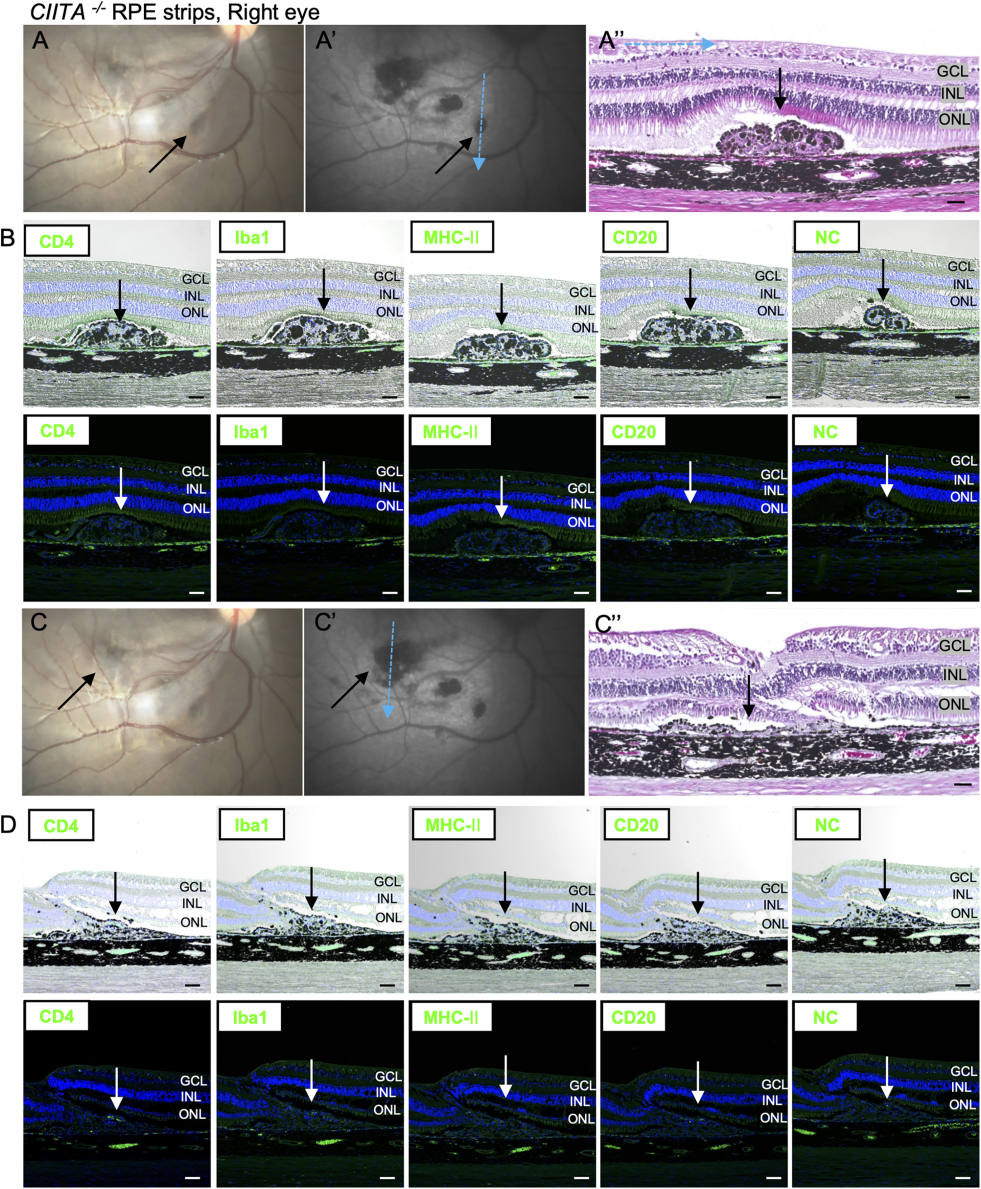
Absence of immune rejection in *CIITA^−^^/^^−^* RPE transplants (monkey 2, right eye). (**A–A**′′, **C–C**′′) Fundus (**A**, **C**) and FAF images (**A**′, **C**′) of *CIITA^−^^/^^−^* RPE transplants in the right eye. Transplanted RPE cells are indicated by *black arrows*. (**A**) Transplanted RPE cells near the arcade vessel. (**C**) Transplanted RPE cells near the fovea. The *blue arrow* denotes the sectioning orientation. H&E staining of *CIITA^−^^/^^−^* RPE strips in the right eye reveals grafted RPE cells (*black arrow*). (**B**, **D**) IHC analysis demonstrates no presence of inflammatory cells (CD4^+^, Iba1^+^, MHC class II^+^, CD20^+^). The *upper panels* show brightfield images overlaid with fluorescent images, and the *lower panels* show fluorescent images. The *black and white arrows* show transplanted RPE cells. NC refers to the negative control with no primary antibody. *Scale bar*: (**A–D**) 50 µm.

**Figure 5.**
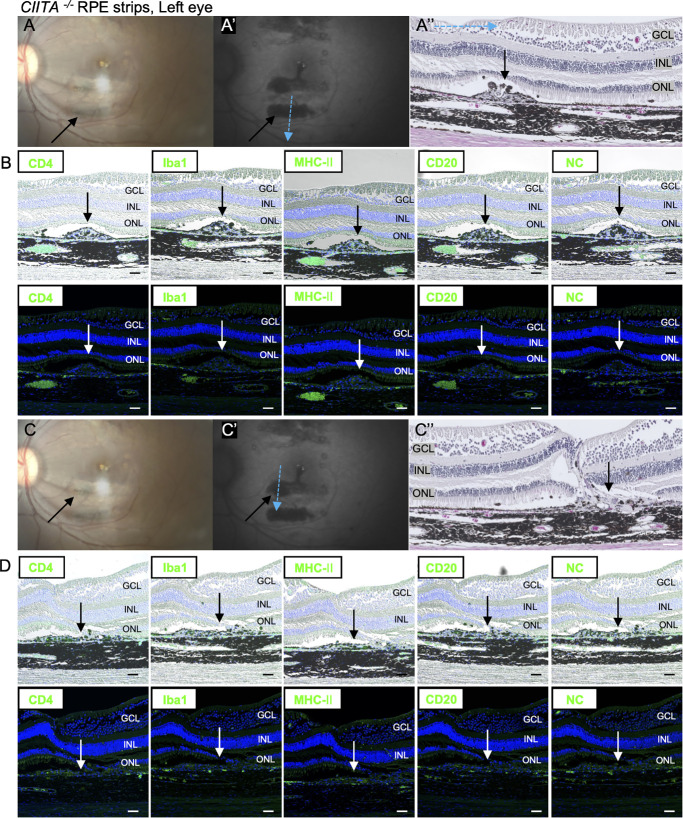
Lack of immune rejection in *CIITA^−^^/^^−^* RPE grafts (monkey 2, left eye). (**A–A**′′, **C–C**′′) Fundus (**A**, **C**) and FAF images (**A**′, **C**′) of *CIITA^−^^/^^−^* RPE transplants in the left eye. Transplanted RPE cells are indicated by *black arrows*. (**A**) Transplanted RPE cells near the arcade vessel. (**C**) Transplanted RPE cells near the fovea. The *blue arrow* indicates the sectioning orientation. H&E staining of *CIITA^−^^/^^−^* RPE strips in the left eye highlights grafted RPE cells (*black arrow*). (**B**, **D**) IHC analysis shows no detectable inflammatory cells (CD4^+^, Iba1^+^, MHC class II^+^, CD20^+^). The *upper panels* present brightfield images overlaid with fluorescence images, while the *lower panels* show fluorescence images. *Black and white arrows* indicate transplanted RPE cells. NC represents the negative control with no primary antibody. *Scale bar*: (**A–D**) 50 µm.

At first, the immune response was evaluated after laser treatment. Iba1-positive macrophage/microglial cells were observed in the choroid close to the damaged RPE cells at 2 days after laser treatment, but they had disappeared by 5 months after laser treatment (Supplementary Fig. S3). These results suggest that the disruption of the blood–retinal barrier alone does not trigger evident inflammatory responses. Next, we evaluated the presence of immune cells at the graft site in the eyes sampled 5 months after transplantation of the second eye. In monkey 1, hematoxylin and eosin staining revealed accumulated immune cells at transplanted *CIITA^+/+^* RPE strips mingled with pigmented cells, possibly graft RPE cells (Fig. 3A′′). Accumulated cells included CD4-, CD8-, IFN-γ-, Iba1-, MHC class Ⅱ–, and CD20-positive immune cells in both the right eye (Fig. 3B, Supplementary Fig. S4A) and the left eye (Fig. 3D, Supplementary Fig. S4B). In monkey 1, the sign of rejection was severe in both eyes, making it difficult to identify individual *CIITA^+/+^* RPE strips. In contrast, monkey 2, which received two CIITA/RPE strips in each eye, showed almost no immune cells, similar to the negative control (Figs. 4, 5, Supplementary Figs. S4C, S4D). These findings indicate that eyes transplanted with *CIITA^−^^/^^−^* RPE strips exhibited no immune rejection by immunohistochemistry (IHC), presenting a clear difference from those with *CIITA^+/+^* RPE strips.

### Engrafted *CIITA*^−/−^ RPEs Exhibited Correct Polarity and RPE Markers

**Figure 6.**
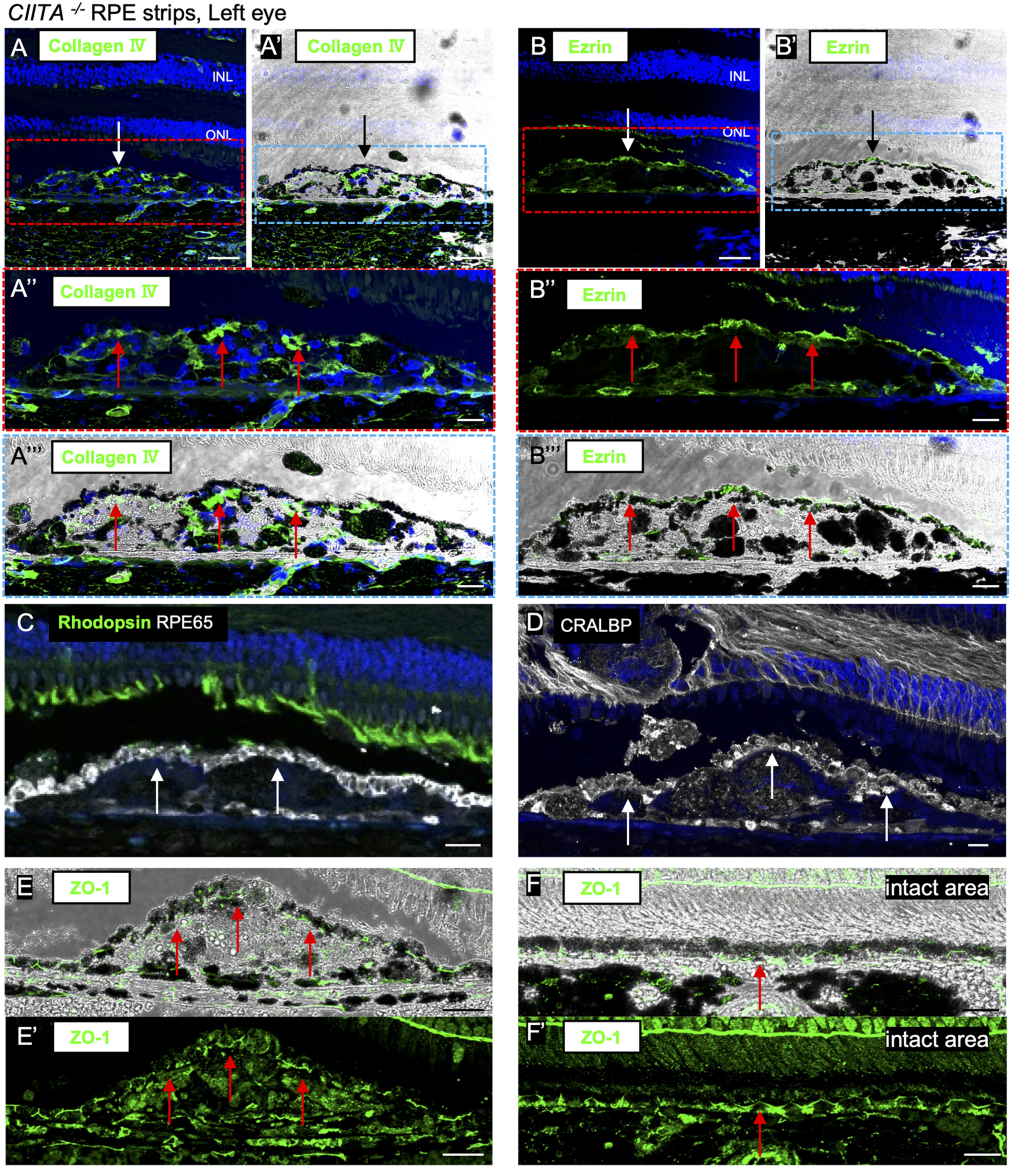
Proper polarity and visual cycle protein expression in *CIITA^−^^/^^−^* grafts of the left eye. (**A–A′′′**, **B–B′′′**) *CIITA^−^^/^^−^* RPE strips (*white and black arrows*) transplanted into the left eye expressed collagen IV (basal marker), which was distributed beneath grafted RPE cells on top of the graft mass (**A–A′′′**) and ezrin (apical marker) on the surface (**B–B′′′**) (*red arrows*). Fluorescent images (**A**, **A′′**, **B**, **B′′**) and brightfield images (**A′**, **A′′′**, **B′**, **B′′′**) are shown, with **A′′**, **A′′′**, **B′′**, and **B′′′** showing enlarged views. (**C**) *CIITA^−^^/^^−^* RPE strips (*white arrows*) in the left eye expressed RPE65. Rhodopsin expression was maintained in rod photoreceptor outer segments above the graft RPE. (**D**) *CIITA^−^^/^^−^* RPE strips (*white arrows*) in the left eye expressed CRALBP. (**E**, **E′**) *CIITA^−^^/^^−^* RPE strips in the left eye expressed Zo-1. Ezrin was expressed on top of the graft mass in the transplanted RPE cells (*red arrows*). Brightfield images (**E**) and fluorescent images (**E′**) are shown. (**F**, **F′**) ZO-1 was expressed in the intact RPE cells (*red arrow*). Brightfield images (**F**) and fluorescent images (**F′**) are shown. *Scale bar*: (**A**, **A′**, **B**, **B′**) 50 µm, (**A′′**, **A′′′**, **B′′**, **B′′′**, **F**, **F′**) 20 µm, (**C**) 10 µm, (**D**) 15 µm, (**E**, **E′**) 30 µm. OS, outer segment.

Interestingly, with *CIITA^−^^/^^−^* RPE grafts, although one strip in the right eye showed a round graft arrangement while the other strips showed flat-on-the edge arrangement, the pigmented RPE monolayer was consistently observed atop the graft mass with the underlying mass consisting of depigmented cells and some pigmented clumps (Fig. 4). In the top RPE monolayer, collagen Ⅳ (basal side marker) was observed basal to the RPE cell layer, and ezrin (apical side marker) was located on the surface of these RPE cells (Figs. 6A–B′′′, Supplementary Figs. S5A–B′′′). The *CIITA^+/+^* graft was also examined, but polarity markers were not expressed (Supplementary Figs. S6A–B′). Another intriguing feature was that, although RPE cells generally exhibit marked autofluorescence in IHC images, RPE cells in the graft overall showed low autofluorescence, likely due to low lipofuscin accumulation (Supplementary Fig. S7), which seemed to help identify the grafted RPEs. Rhodopsin expression was localized in the outer segments of rod photoreceptor cells above the *CIITA^−^^/^^−^* RPEs, similar to healthy photoreceptor cells, whereas it was distributed throughout the rod photoreceptor cell membrane above the *CIITA^+/+^* graft with few RPE-like cells, indicating the stress on host rod photoreceptor cells (Fig. 6C, Supplementary Figs. S5C, S6D–D′′). Furthermore, RPE65 and CRALBP, visual cycle-related proteins, were expressed on the surface of *CIITA^−^^/^^−^* RPE cells, suggesting their functionality, while there were no RPE65- and CRALBP-positive RPEs in the *CIITA^+/+^* graft mass (Figs. 6C, 6D, Supplementary Figs. S5C, S5D, S6C–D′′). Next, we evaluated the tight junction in the transplanted RPE cells by immunolabeling ZO-1. ZO-1 was expressed at the border of the cells in the top RPE layer of the *CIITA^−^^/^^−^* graft mass (Figs. 6E, 6E′, Supplementary Figs. S5E–F′), similar to intact RPE monolayer cells (Figs. 6F–F′). Finally, we tried characterizing the graft mass beneath the correctly aligned RPE layer, which was seemingly losing RPE properties. The cells in the graft mass did not express caspase 3 or Ki67, in contrast to the cells in the *CIITA^+/+^* graft mass that include many infiltrating proliferating immune cells as well as the cells under apoptosis (Supplementary Figs. S8A–D′). Notably, α-SMA–positive cells were not detected in the *CIITA^−^^/^^−^* graft, indicating that the mass did not likely include the cells under epithelial–mesenchymal transition (Supplementary Figs. S8H, S8H′). Instead, the cells in the mass were positive for GFAP but negative for GS and CRALBP (Supplementary Figs. S8E–G′), indicating the potential infiltration of astrocytes or Müller cells that are losing innate properties in the graft mass. Additionally, GFAP staining in the host retina suggested moderate activation of Müller cells at 5 months after laser treatment, regardless of RPE engraftment. This activation was not evident above the RPE strips that extended beyond the lasered area into the nonlasered area, suggesting that RPE graft alone may not enhance Müller glia activation (Supplementary Figs. S8I).

## Discussion

In this study, we transplanted allogenic *CIITA^−^^/^^−^* and *CIITA^+/+^* RPE strips into MHC-mismatched monkey eyes with RPE damage without using systemic immune suppression. We observed no immune response in monkey eyes transplanted with *CIITA^−^^/^^−^* RPE strips, even after repeated transplantation of both eyes, while evident immune rejections occurred with the *CIITA^+/+^* graft. Additionally, *CIITA^−^^/^^−^* RPEs showed expansion in the grafted area and, interestingly, were often present with correct apical–basal polarity on top of the remaining graft mass. These *CIITA^−^^/^^−^* RPEs expressed visual cycle-related protein RPE65, and overlying photoreceptors retained rhodopsin-positive outer segments, while *CIITA^+/+^* RPE cells seemed to be losing their functional properties due to immune rejection. This may leave important questions as to whether these cells can still recover their RPE properties with some intervention such as steroid treatment and, critically, what the optimal timing window for such an intervention would be before reaching a “point of no return” in the rejection process.

A previous study showed that hiPSC-RPE suspensions lacking MHC class Ⅰ, Ⅱ, or both demonstrated delayed rejection compared to nonedited grafts when transplanted into rabbit eyes (xenogeneic transplantation), although no significant differences were observed among the different MHC knockout genotypes.^9^ MHC class Ⅰ knockout reduces the immune response of CD8-positive T cells but increases the risk of NK-mediated lysis, by activating the missing-self response.^16^ NK-cell degranulation was higher in MHC class Ⅰ–deficient hiPSC-RPE cells compared to that in nonedited RPE cells in vitro.^9^ To counteract NK-mediated lysis, strategies such as retaining HLA-C or expressing HLA-E or G in MHC class Ⅰ knockout cells have been proposed.^17^^–^^19^ However, their efficacy in suppressing NK cells after transplantation remains unclear. Additionally, MHC class Ⅰ knockout increases the risk of viral infection and tumorigenesis. Our attempt to solely delete MHC class II, therefore, proves to be an advantageous strategy.

Our present question was whether we could substantially suppress rejection by MHC class Ⅱ knockout alone in RPE cell transplantation. We previously showed that allogeneic MHC class Ⅱ–deficient moiPSC-RPE suspensions reduced immune response compared to nonedited grafts in vitro and potentially in vivo. However, the rejection of nonedited grafts was also mild, possibly due to the scattered engraftment of RPE suspensions. In this study, nonedited grafts served as a positive control exhibiting a marked rejection from 1 to 2 months after transplantation in both eyes, while allogeneic MHC class Ⅱ–deficient moiPSC- RPE strips showed no signs of rejection in either eye for 5 months without systemic immunosuppression. This was consistent with our previous observation that the moESC-RPE sheet lacking MHC class II did not cause any immune rejection in allogenic monkey transplantation.^11^ Furthermore, no rejection occurred despite sequential implantation in both eyes, which is clinically valuable for potential recipients who are likely to have bilateral disease.

With the *CIITA^−^^/^^−^* iPSC-RPE transplant, although MHC class II is deficient even in response to inflammation, immune response by other antigen-presenting cells such as B cells and dendritic cells should remain. RPE cells, including hiPSC-RPE cells, inherently possess immunosuppressive properties, such as the secretion of TGF-β and expression of programmed cell death ligand 1. With these features combined, MHC class Ⅱ deletion in RPEs alone may have resulted in suppressive immune responses against these grafted RPEs.^20^^–^^22^ However, long-term immune responses need further testing, with additional elucidation of essential mechanisms of immunosuppression by MHC class II deletion.

The limitation of this study is that we have only one monkey for each *CIITA^+/+^* and *CIITA^−^^/^^–^* RPE strip group. Therefore, we tried maximizing the immune response by transplanting into both eyes, and the results were quite consistent in the eyes of both monkeys. Also, we could not include lymphocyte–graft cell immune reaction (LGIR) tests in the current study because of a technical issue. However, the rejection patterns and immune response differences between the two monkeys were apparent beyond the use of generally sensitive LGIR.

## Conclusions

In summary, allogeneic transplantation of *CIITA^−^^/^^−^* RPE strips showed no rejection without systemic immunosuppression, even in eyes with the RPE-ablated disease model. Additionally, these grafts demonstrated proper polarity and with one of the visual cycle proteins, while supporting overlying photoreceptor health. This suggests that *CIITA^−^^/^^−^* RPE strips may provide reliable and functional engraftment for clinical applications.

## Supplementary Material

Supplement 1
