# Supplementary material for: Allogenic Transplantation of RPE Strips Lacking MHC Class II Can Avoid Rejection in Nonhuman Primate Eyes
Source: Invest Ophthalmol Vis Sci. 2025 Jun 17;66(6):53. doi: 10.1167/iovs.66.6.53 (PMC12178438; doi:10.1167/iovs.66.6.53)
Supplement: Supplement 1 [file iovs-66-6-53_s001.pdf]

Supplemental information

Allogenic Transplantation of RPE Strips Lacking MHC-Class II Can Avoid Rejection in Non-Human Primate Eyes

Atsuta Ozaki[1,2,3], Sunao Sugita[1,4,5], Masaaki Ishida[6], Mitsuhiro Nishida[1,2,4], Kyoko Iseki[1,2,4], Noriko Sakai[1,4], Naoko Hayashi[1,2,4], Takashi Shiina[7], Satoshi Yokota[1,8], Shin-ichiro Ito[1,8], Masashi Fujihara[1,8], Mineo Kondo[3], Masayo Takahashi[1,2,4], Yasuo Kurimoto[1,8], Michiko Mandai[1,8]

[1] Kobe City Eye Hospital, Hyogo, Japan
[2] Cell and Gene Therapy in Ophthalmology Laboratory, BZP, RIKEN, Wako, Saitama 351-0198, Japan
[3] Department of Ophthalmology, Mie University Graduate School of Medicine, Mie, Japan
[4] Vision Care Inc., Hyogo, Japan
[5] Sugita Eye Clinic, Miyazaki, Japan
[6] Department of Ophthalmology, University of Toyama Graduate School of Medicine and Pharmaceutical Sciences, Toyama, Japan
[7] Tokai University school of Medicine, Kanagawa, Japan
[8] Laboratory for Animal Resources and Genetic Engineering, RIKEN Center for Biosystems Dynamics Research, Hyogo, Japan

Supplemental Figures 1-8
Supplemental Tables 1-3

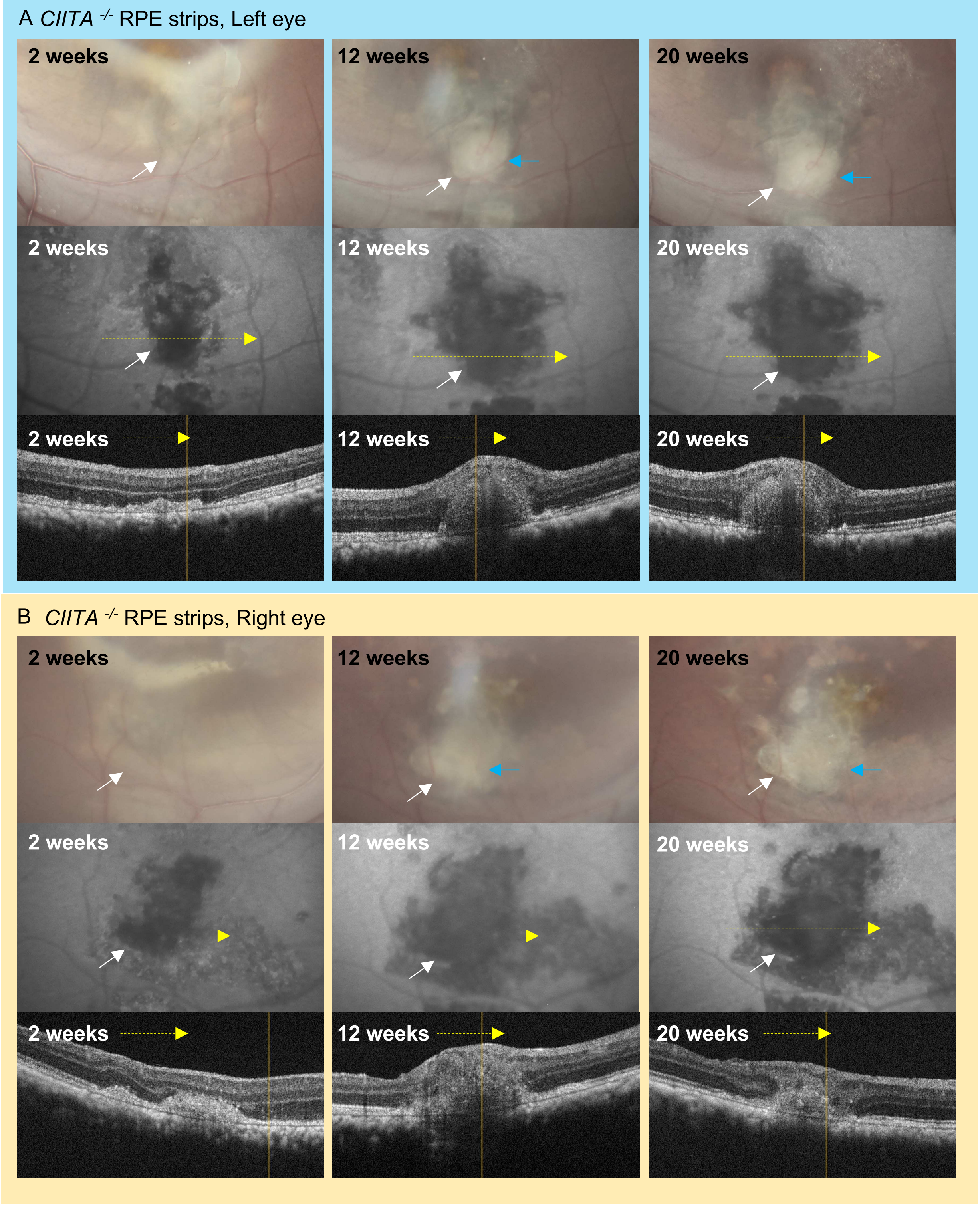

A CIITA -/- RPE strips, Left eye
2 weeks
12 weeks
20 weeks
2 weeks
12 weeks
20 weeks
2 weeks
12 weeks
20 weeks
B CIITA -/- RPE strips, Right eye
2 weeks
12 weeks
20 weeks
2 weeks
12 weeks
20 weeks
2 weeks
12 weeks
20 weeks

Figure S1. Temporal changes in *CIITA* $^{+/+}$ RPE strip transplantation in both eyes

CIITA$^{+/+}$ moiPSC-derived RPE strips were transplanted into Monkey 1. Representative magnified images at 2, 12, and 20 weeks post-transplantation are shown. The transplanted RPE strips, which appeared obscure due to reduced pigmentation (white arrows). White material surrounding the graft site (blue arrows) may indicate immune cell infiltration. On FAF imaging, the RPE engrafted site appeard hypofluorescent potentially by both of immune cell accumulation and the presence of RPE strips. A bulge at the graft site was observed by OCT. Scale bar: 300μm Abbreviation: moiPSC; monkey induced pluripotent stem cell, RPE; retinal pigment epithelium, FAF; fundus autofluorescence, FA; fluorescence angiography, OCT; optical coherence tomography

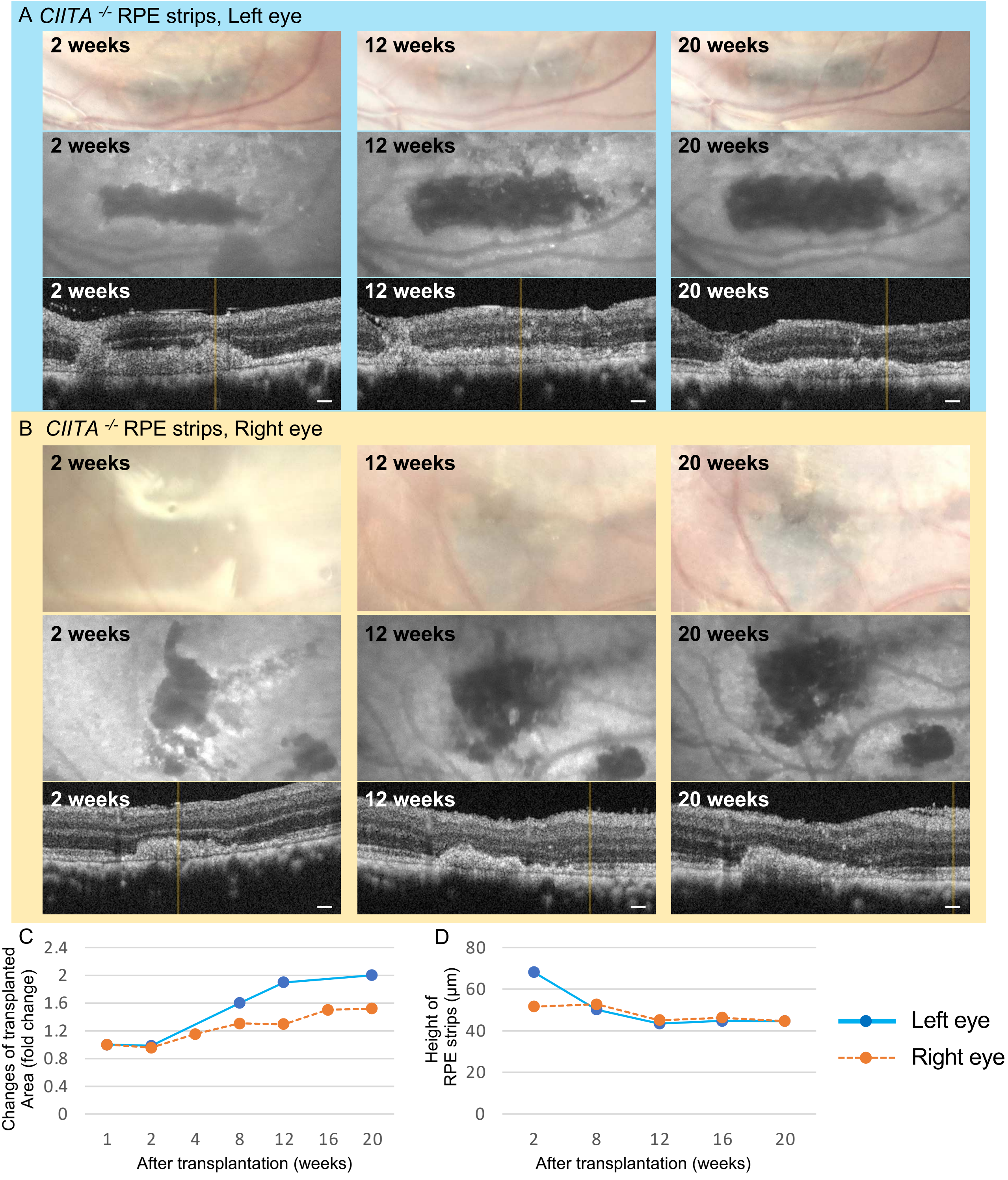

A CIITA -/- RPE strips, Left eye
2 weeks
12 weeks
20 weeks
B CIITA -/- RPE strips, Right eye
C
Changes of transplanted Area (fold change)
2.4
2
1.6
1.2
0.8
0.4
0
1 2 4 8 12 16 20
After transplantation (weeks)
D
Height of RPE strips (μm)
80
60
40
20
0
2 8 12 16 20
After transplantation (weeks)
Left eye
Right eye

Figure S2. Temporal changes in engrafted RPE strip transplantation.

(A, B) Serial changes in the engrafted area following *CIITA*$^{-/-}$ RPE strip transplantation in the left (A) and right (B) eyes. Representative images are shown at 2weeks, 12weeks, and 20weeks post-transplantation. Increase in pigmentation was observed over time. (C) Quantification of the graft area are normalized to the baseline measurement at 1week post-transplantation. Graft areas, as visualized by FAF, were manually delineated at the engraftment site using ImageJ software. The *CIITA* $^{-/-}$ RPE strips demonstrated progressive expansion over time in both eyes. (D) Quantification of maximal *CIITA* $^{-/-}$ RPE strip height in each eye. The height of the RPE strips progressively decreased over time.

Scale bar: (A-B) 150μm

Abbreviation: RPE; retinal pigment epithelium, FAF; fundus autofluorescence, OCT; optical coherence tomography

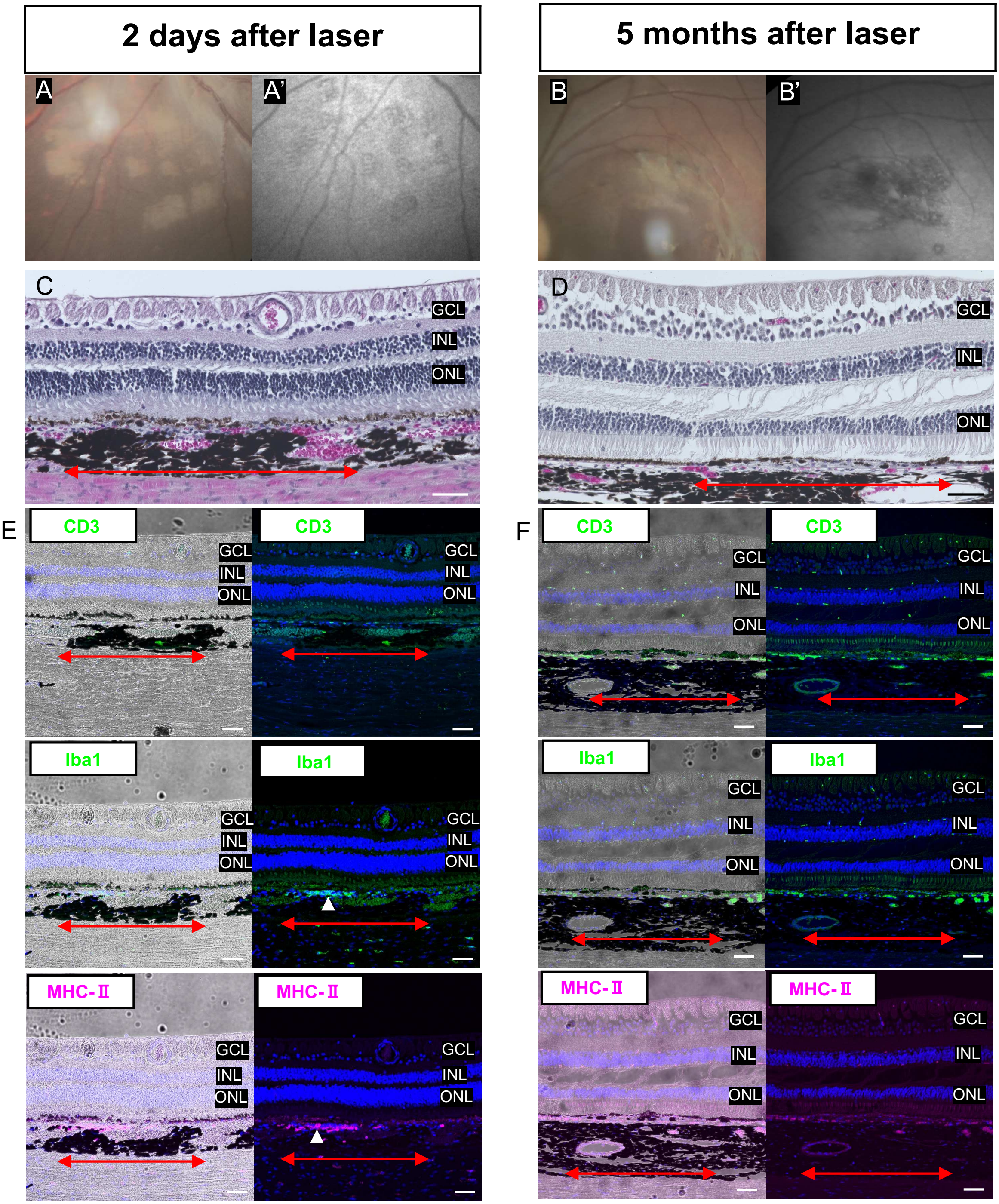

2 days after laser
5 months after laser
A
A'
B
B'
C
D
GCL
INL
ONL
E
F
CD3
Iba1
MHC-Ⅱ

Figure S3. Changes in Immune Cell Accumulation at 2 Days and 5 Months After Micropulse Laser Treatment

(A-B') Fundus (A, B) and FAF (A', B') images at 2 days and 5 months after micropulse laser treatment. (C, D) H&E staining of the laser-treated area (red double-headed arrow). (E) IHC analysis showed no detectable CD3+ cells, while Iba1+ and MHC class II cells (DAPI positive cells) with accumulated between the RPE and choroidal vessels (white arrowheads). (F) IHC analysis demonstrated no presence of inflammatory cells (CD3+, Iba1+, MHC class II). The paraffin sections were obtained from the monkey eye that had received the same microsecond pulse laser treatment with the current protocol in our previous report[2]

Scale bar: (C-F) 50µm

Abbreviation: FAF; fundus autofluorescence, H&E; hematoxylin and eosin, IHC; immunohistochemistry, CD; cluster of differentiation

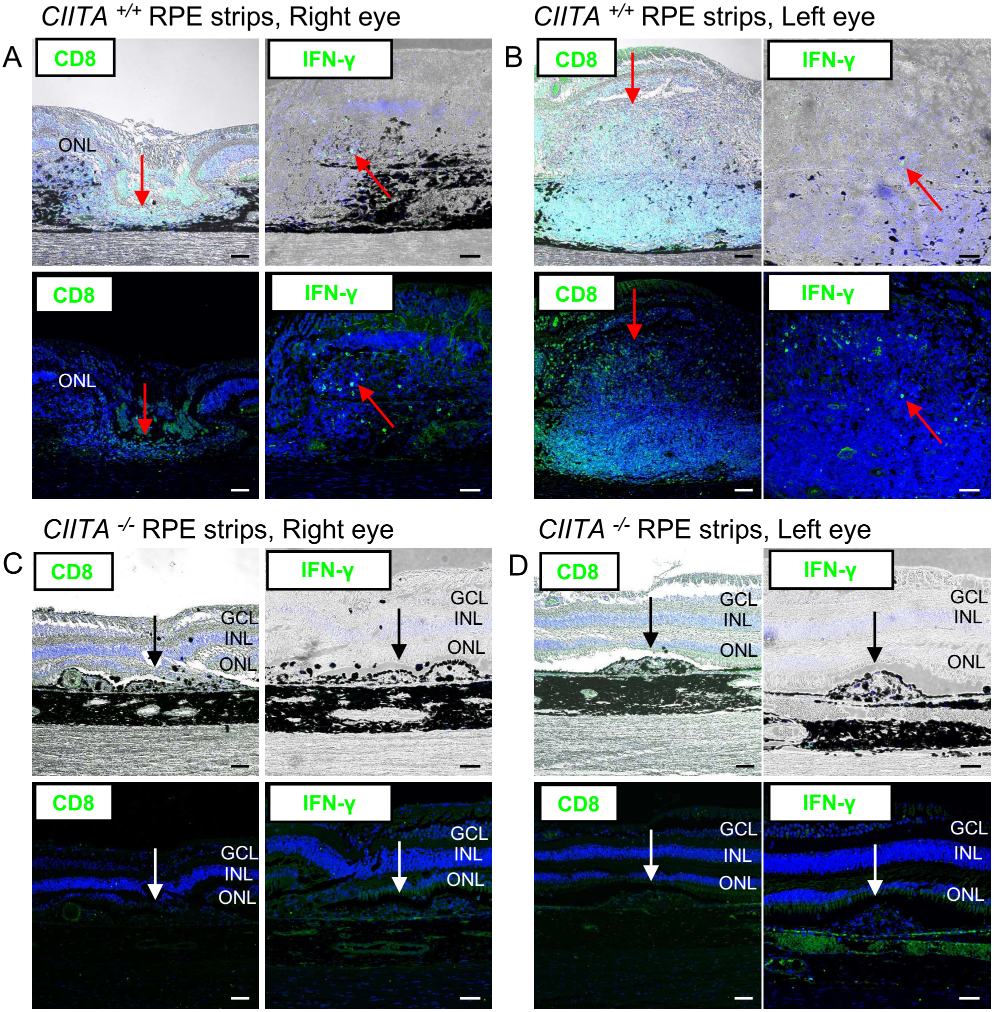

Figure S4. IHC analysis of inflammatory cell presence in *CIITA* $^{+/+}$ RPE grafts

(A, B) IHC analysis revealed the presence of inflammatory cells (CD8+, INFγ+) in the right (A) and left (B) eyes of the *CIITA* $^{+/+}$ RPE grafts. The upper panels show brightfield images overlaid with fluorescent images, while the lower panels show fluorescent images. The red arrows indicate accumulated immune cells. (C, D) IHC analysis showed no detectable inflammatory cells. Transplanted RPE cells are indicated by black and white arrows.

Scale bar: (A-D) 50μm Abbreviation: IHC; immunohistochemistry, RPE; retinal pigment epithelium, CD; cluster of differentiation

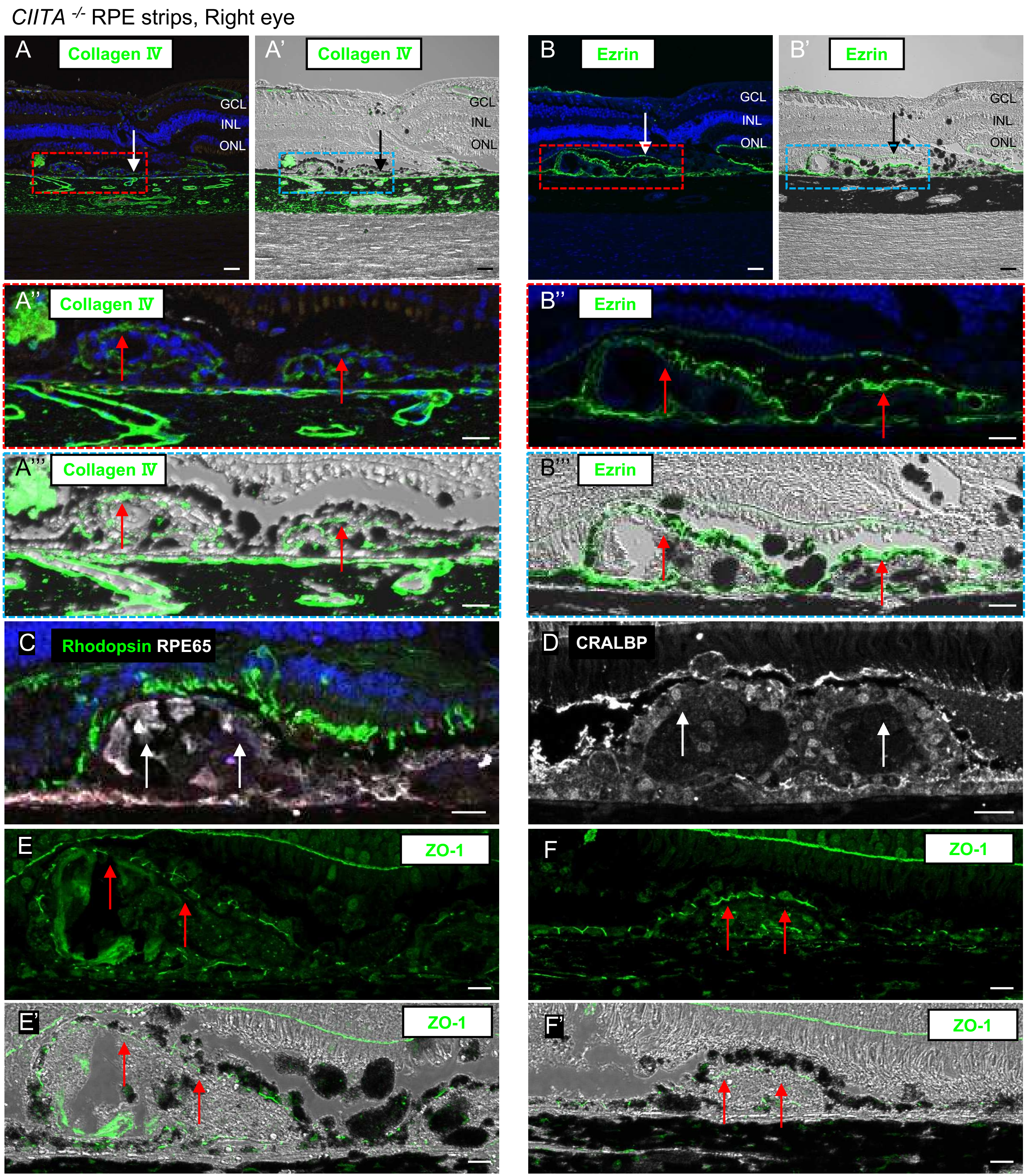
CIITA -/- RPE strips, Right eye
A
Collagen IV
GCL
INL
ONL
A'
Collagen IV
GCL
INL
ONL
B
Ezrin
GCL
INL
ONL
B'
Ezrin
GCL
INL
ONL
A''
Collagen IV
B''
Ezrin
A'''
Collagen IV
B'''
Ezrin
C
Rhodopsin RPE65
D
CRALBP
E
ZO-1
F
ZO-1
E'
ZO-1
F'
ZO-1

Figure S5. Proper polarity and visual cycle protein expression in *CIITA$^{-/-}$* grafts of the right eye
(A-A''', B-B''') *CIITA$^{-/-}$* RPE strips (white and black arrows) transplanted into the right eye exhibited Collagen IV (basal marker) localized beneath the grafted RPE cells on top of graft mass (A-A''') and Ezrin (apical marker) expressed on the surface (B-B''') (red arrows). Fluorescent images (A, A'', B, B'') and brightfield images (A', A''', B', B''') are shown, with A'', A''', B'' and B’’’ representing magnified views. (C) *CIITA $^{-/-}$* RPE strips (white arrows) in the right eye expressed RPE65. Rhodopsin expression was maintained in rod photoreceptor outer segments above the graft RPE. (D) *CIITA $^{-/-}$* RPE strips (white arrows) in the right eye expressed CRALBP. (E, E’, F, F’) *CIITA $^{-/-}$* RPE strips in the right eye expressed ZO-1. ZO-1 was expressed on top of graft mass (red arrows). Brightfield images (E’, F’) and fluorescent image (E, F) are shown.
Scale bar: (A, A’, B, B’) 50μm, (A’’, A’’’, B’’, B’’’) 20μm, (C) 10μm, (D) 25μm, (E-F’) 15μm
Abbreviation: RPE; retinal pigment epithelium

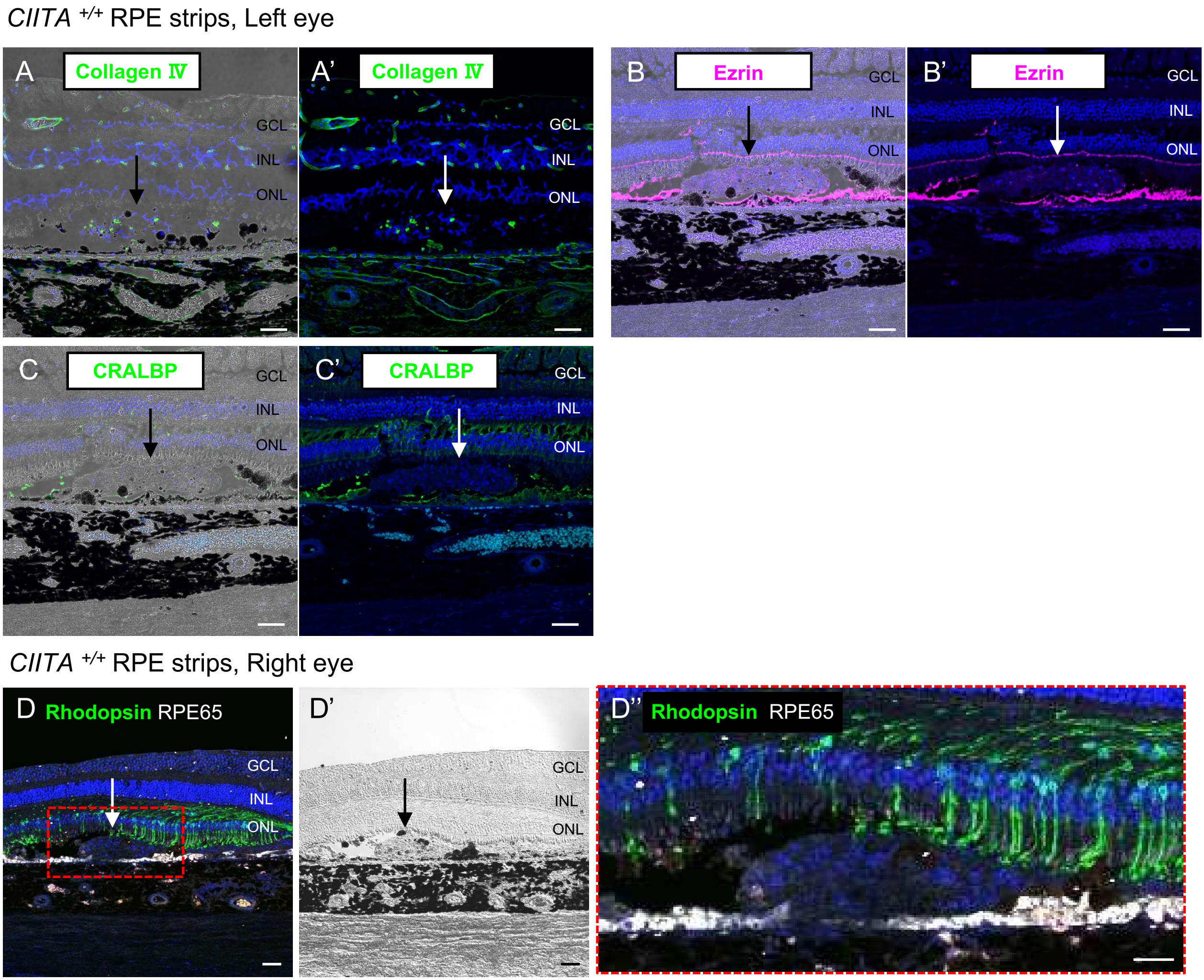

Figure S6. Absence of polarity and RPE marker expression in *CIITA*$^{+/+}$ grafts

(A-C') *CIITA*$^{+/+}$ RPE strips (white and black arrows) did not exhibit expression of Collagen IV (basal marker), Ezrin (apical marker), and CRALBP (visual cycle marker). Fluorescent images (A', B', C') and brightfield images (A, B, C) are shown (D-D'') *CIITA*$^{+/+}$ RPE strips (white and black arrows) in the right eye showed no RPE65 expression. Rhodopsin expression was abnormally distributed throughout the rod cell bodies above the *CIITA*$^{+/+}$ graft. Fluorescent images (D, D'') and brightfield image (D') are shown, with (D'') representing a magnified view.

Scale bar: (A-D') 50μm, (D'') 10μm

Abbreviation: RPE; retinal pigment epithelium,

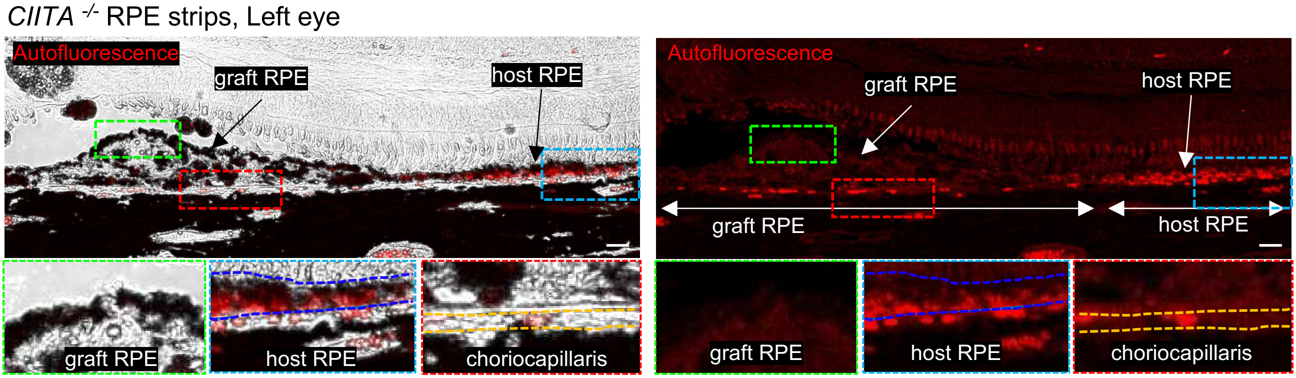

Figure S7. Distinction of grafted and host RPE cells based on autofluorescence intensity

Grafted RPE cells were distinguishable from host RPE cells based on autofluorescence intensity, with grafted cells (green dotted box with magnification) showing minimal autofluorescence compared to the distinct autofluorescence of host RPE cells (blue dotted box with magnification). Red dotted box shows autofluorescence in choriocapillaris.

Scale bar: 25μm

Abbreviation: RPE; retinal pigment epithelium

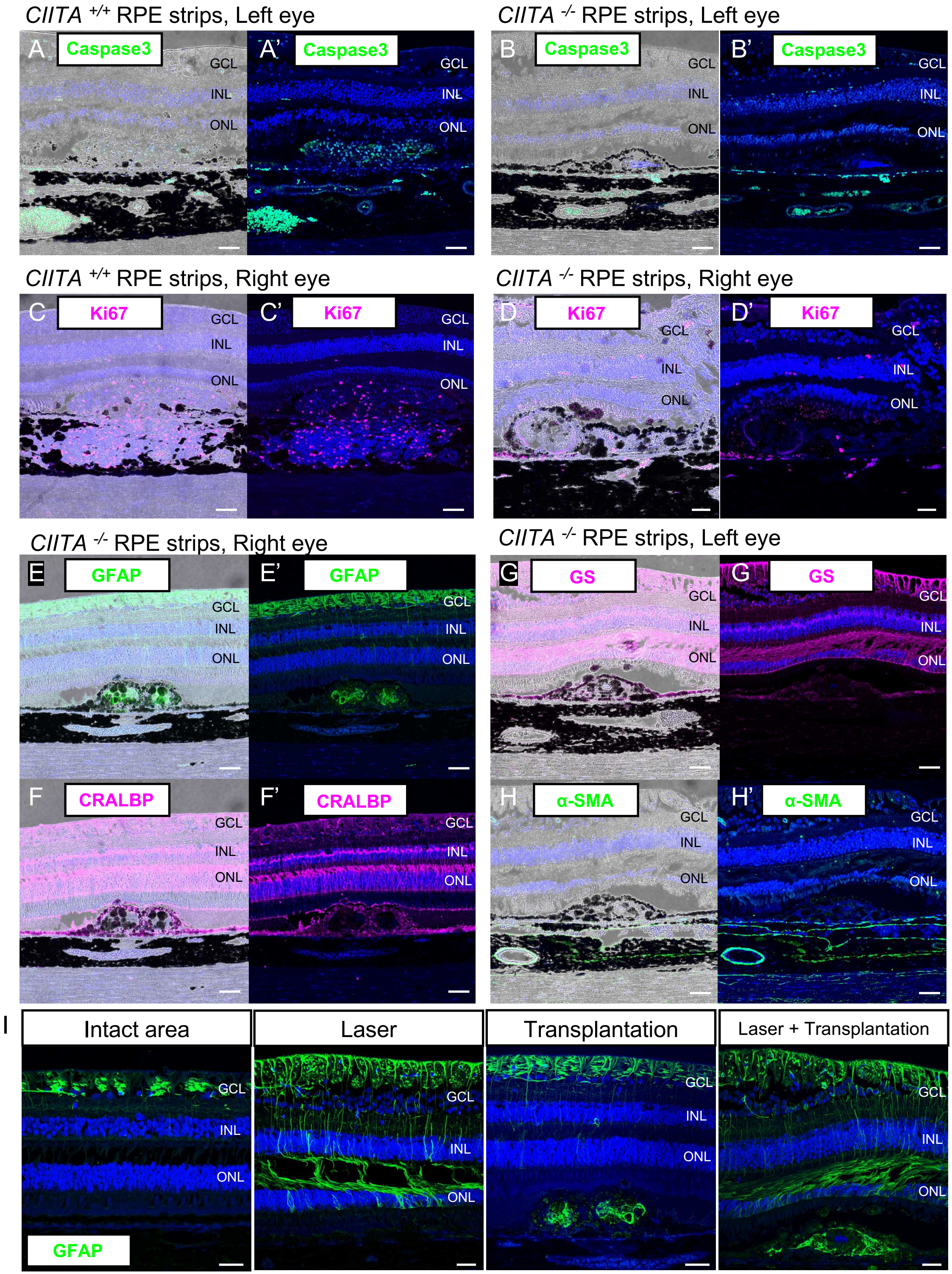
CIITA +/+ RPE strips, Left eye
CIITA -/- RPE strips, Left eye
A Caspase3
A' Caspase3
B Caspase3
B' Caspase3
CIITA +/+ RPE strips, Right eye
CIITA -/- RPE strips, Right eye
C Ki67
C' Ki67
D Ki67
D' Ki67
CIITA -/- RPE strips, Right eye
CIITA -/- RPE strips, Left eye
E GFAP
E' GFAP
G GS
G GS
F CRALBP
F' CRALBP
H α-SMA
H' α-SMA
GCL
INL
ONL
I
Intact area
Laser
Transplantation
Laser + Transplantation
GFAP

Figure S8. The underlying mass beneath the RPE strips include GFAP positive cells

(A, A’) Cleaved caspase-3-positive apoptotic cells were observed within the *CIITA*$^{+/+}$ RPE graft. (B, B’) Cleaved caspase-3-positive cells were not detected within the *CIITA*$^{-/-}$ RPE graft. (C, C’) Ki67-positive proliferating cells were observed within the *CIITA*$^{+/+}$ RPE graft. (D, D’) Ki67-positive cells were not detected within the *CIITA*$^{-/-}$ RPE graft. (E–E’) The underlying mass beneath the top RPE monolayer contained GFAP-positive cells.
(F-G’) Cells beneath the RPE strips were negative for Müller cell marker GS and CRALBP.
(H, H’) The underlying mass beneath the top RPE monolayer lacked α-SMA-positive myofibroblast cells. (I) Comparison of GFAP expression in the intact area, 5 months after laser treatment, after transplantation extending over the non-lasered area, and transplantation after laser treatment. Scale bar: (A-C’, E-H’) 50μm, (D, D’, I) 30μm Abbreviation: RPE; retinal pigment epithelium

Table S1. Summary of monkeys transplanted with RPE strips

Monkey number	Monkey 1		Monkey 2	
Age at transplantation	4 years old		5 years old	
Operate eye	Right	Left	Right	Left
The number of RPE strip	2	2	2	2
RPE type	$CIITA^{+/+}$	$CIITA^{+/+}$	$CIITA^{-/-}$	$CIITA^{-/-}$
Rejection sign in vivo	+	+	-	-
Immune cells by IHC	+	+	-	-

Table S2. Major Histocompatibility Complexes of monkeys (Mafa)

	Monkey 1	Monkey2	1121A1
**Mafa-A**	A1*097:01	A1*066:06/07	A1*052:02
		A1*003:06	A4*01:04
		A4*14:03/04	A8*08:01
**Mafa-B**	B*056:01	B*030:15	B*033:02
	B*007/05/08/10	B*105:01	B*095:01
	B*045:06/09	B*156:01	B*098:10
	B*193:01	B*060:33	
	B*032:03	B*051:05/08	
**Mafa-DRB**	DRB1*04:07	DRB1*03:03/30	DRB1*03:21
	DRB*W003:08	DRB1*04:07	DRB1*10:07
	DRB*W007:10	DRB*W001:01	
	DRB*W021:01	DRB*W003:02	
	DRB*W027:01	DRB*W003:08	
	DRB1*04:07:02	DRB*W007:10	
**Mafa-DQB1**	DQB1*15:03	DQB1*06:19	DQA1*01:07:01
	DQB1*28:01	DQB1*06:27	DQB1*06:08
**Mafa-DPB1**	DPB1*01:01/07/13	DPB1*15:01/13/14	DPA1*02:05
	DPB1*02:01	DPB1*16:01	DPB1*15:04

Table S3. List of primary antibodies

Primary Antibody	host	Dilution	Catalog#	Source
**α-SMA**	Rb	1:100	ab5694	Abcam
**CD3**	Rb	1:100	Ab16669	Abcam
**CD4**	Rb	1:100	ab133616	Abcam
**CD8**	Rb	1:500	Ab4055	Abcam
**CD20**	Rb	1:100	ab78237	Abcam
**Cleaved-Caspase3**	Rb	1:400	9661T	Cell signaling
**Collagen4**	Ms	1:400	Ab6311400	Abcam
**CRALBP**	Ms	1:200	Ab15051	Abcam
**Ezrin**	Rb	1:1000	MAB72391000	R &D Systems
**GFAP**	Rb	1:500	Z0334-29-2J	Dako
**GS**	Ms	1:1000	MAB302	Millipore
**HLA-DP, DQ, DR**	Ms	1:100	M0775	Dako
**Iba1**	Rb	1:1000	019-19741	Wako
**IFNγ**	Ms	1:150	MAB2851	R&D systems
**Ki67**	Ms	1:200	550609	BD Biosciences
**Rhodopsin**	Ms	1:500	O4886500	Sigma
**RPE65**	Rb	1:1000	AS23521000	Takara
**Zo-1**	Rb	1:200	61-7300	Invitrogen
